# A new species of *Brachycephalus* (Anura: Brachycephalidae) from the Quiriri mountain range of southern Brazil

**DOI:** 10.7717/peerj.1179

**Published:** 2015-08-13

**Authors:** Marcio R. Pie, Luiz F. Ribeiro

**Affiliations:** 1 Laboratório de Dinâmica Evolutiva e Sistemas Complexos, Departamento de Zoologia, Universidade Federal do Paraná, Centro Politécnico, Curitiba, Paraná, Brazil; 2 Mater Natura—Instituto de Estudos Ambientais, Curitiba, Paraná, Brazil; 3 Faculdade Dom Bosco, Avenida Presidente Wenceslau Braz, Curitiba, Paraná, Brazil

**Keywords:** Amphibia, Atlantic Rainforest, Cloud forest, Terrarana, Serra do Quiriri

## Abstract

A new miniaturized toadled of the genus *Brachycephalus* (Anura: Brachycephalidae) is described from Serra do Quiriri in the municipality of Campo Alegre, Santa Catarina, southern Brazil. Specimens were collected from the leaf litter between from 1,263 and 1,318 m above sea level. The new species is distinguished from all its congeners by the combination of the following characters: snout–vent length 9.9–13.1 mm; skin on head and dorsum without dermal co-ossification; snout mucronate in dorsal view; dorsum rugose; general color brown, with a narrow orange vertebral stripe. The region where the new species is located is also shared with other endemic anuran species and has experienced strong anthropogenic impacts,suggesting that immediate actions should be taken to ensure their long-term preservation.

## Introduction

The Brazilian Atlantic Forest (BAF) is known to harbor exceptionally high species richness, being considered as one of the world’s biodiversity hotspots (Myers et al., 2000). However, the distribution of both species and genetic diversity is uneven throughout the BAF (e.g., Da Silva, Cardoso de Sousa & Castelletti, 2004; Sigrist & Carvalho, 2008), possibly as a result of the complex evolutionary history of the biome as it responded to climatic changes over the course of the Quaternary (Carnaval et al., 2009; Carnaval et al., 2014). Some taxa endemic to the BAF show extremely reduced geographical ranges, most commonly in association with montane regions, such as many species of the miniaturized toadlet *Brachycephalus* Fitzinger, 1826 (Anura: Brachycephalidae) (Pombal, Wistuba & Bornschein, 1998; Ribeiro et al., 2005; Alves et al., 2006; Napoli et al., 2011; Pie et al., 2013). Despite the intriguing evolutionary mechanisms underlying the diversification of *Brachycephalus*, only recently have their phylogenetic relationships begun to be investigated (e.g., Clemente-Carvalho et al., 2011; Padial, Grant & Frost, 2014).

The species of *Brachycephalus* have been recently divided into three species groups (Ribeiro et al., 2015): *ephippium* (Spix, 1824), *didactylus* (Izecksohn, 1971), and *pernix* Pombal, Wistuba & Bornschein, 1998. The latter included the species of *Brachycephalus* that shared their bufoniform body shape with the *ephippium* group, but that lacked their characteristic dermal ossification, namely *B. pernix* Pombal, Wistuba & Bornschein, 1998, *B. brunneus* Ribeiro, Alves, Haddad & Reis, 2005, *B. izecksohni* Ribeiro, Alves, Haddad & Reis, 2005, *B. ferruginus* Alves, Ribeiro, Haddad & Reis, 2006, *B. pombali* Alves, Ribeiro, Haddad & Reis, 2006, and *B. tridactylus* Garey, Lima, Hartmann & Haddad, 2012. However, a single additional study more than doubled the number of species in the *pernix* group (Ribeiro et al., 2015), suggesting that the actual diversity in the group could be greatly underestimated. In the present study, we describe a new species of the *pernix* group of *Brachycephalus* from the Quiriri mountain range of the state of Santa Catarina, southern Brazil. In addition to clear morphological diagnostic traits, the new species tends to be isolated from one another by valleys of unsuitable habitats, essentially forming sky islands (Howell, 1947; see McCormack, Huang & Knowles, 2009 for a recent review).

## Materials and Methods

Adult specimens were anaesthetized and euthanized using 2% lidocaine, fixed in 10% formalin, and preserved in 70% ethanol. Measurements were made with a micrometric eyepiece attached to a stereomicroscope. Measurements abbreviations were as follows: snout-vent length (SVL); head length, from tip of snout to angle of jaw (HL); head width—greatest width of head located between angles of jaw (HW); eye diameter (ED); nostril diameter (ND); interorbital distance between anterior corners of the eyes (IOD); internostril distance between inner margins of nostrils (IND); eye-nostril distance from anterior corner of the eye to posterior margin of nostril (END); thigh length (THL); and tibia length (TBL).

All specimens collected for this study were deposited in the Coleção Herpetológica do Departamento de Zoologia, Universidade Federal do Paraná, Curitiba, Paraná, Brazil (DZUP). In addition, we examined specimens from the following collections: Célio F. B. Haddad collection, deposited in the Departamento de Zoologia, Universidade Estadual Paulista, Campus de Rio Claro, São Paulo, Brazil (CFBH), DZUP, Museu de História Natural Capão da Imbuia, Curitiba, Paraná, Brazil (MHNCI), Museu Nacional, Rio de Janeiro, Rio de Janeiro, Brazil (MNRJ), Museu de Zoologia da Universidade de São Paulo, São Paulo, São Paulo, Brazil (MZUSP), and Museu de História Natural, Universidade Estadual de Campinas, Campinas, São Paulo, Brazil (ZUEC). A list of the examined specimens is provided in Appendix S1. In this study we adopted the evolutionary species concept, which equates species with “separately evolving (segments of) metapopulation lineages” (De Queiroz, 2005), which is particularly suitable for the evolutionary scenario of population isolation in sky islands followed by divergent evolution.

The electronic version of this article in Portable Document Format (PDF) will represent a published work according to the International Commission on Zoological Nomenclature (ICZN), and hence the new name contained in the electronic version is effectively published under that Code from the electronic edition alone. This published work and the nomenclatural act it contains has been registered in ZooBank, the online registration system for the ICZN. The ZooBank LSIDs (Life Science Identifiers) can be resolved and the associated information viewed through any standard web browser by appending the LSID to the prefix “http://zoobank.org/.” The LSID for this publication is: 5267B6A8-373D-4DD2-9F7F-9D41FB6933E0. The online version of this work is archived and available from the following digital repositories: PeerJ, PubMed Central and CLOCKSS.

Collection permits for this study were issued by ICMBIO (SISBIO 1911426). We classified vegetation in collection sites according to the classification of Veloso, Rangel-Filho & Lima (1991). All coordinates in this study were based on datum WGS84.

**Figure 1:**
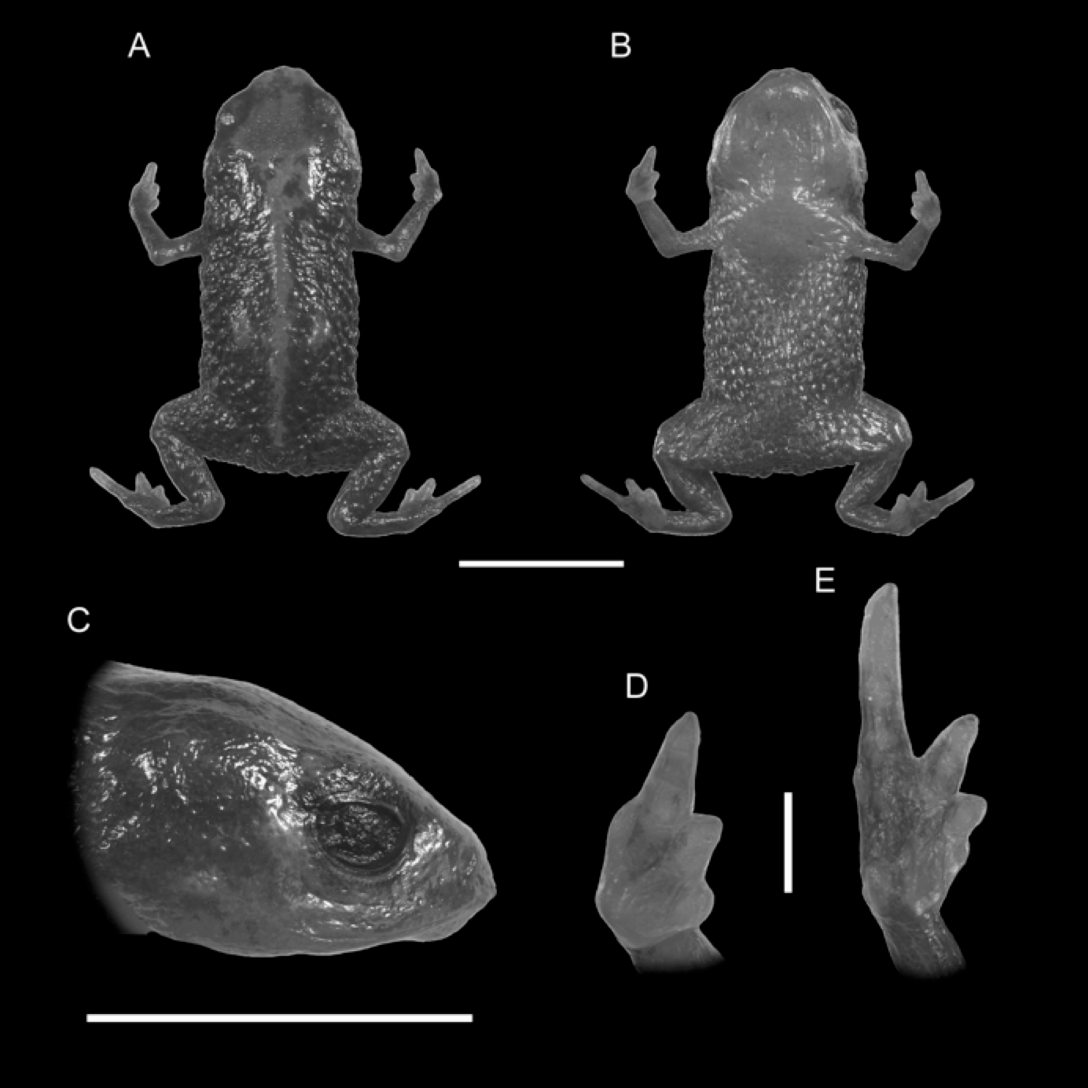
Holotype of *Brachycephalus quiririensis* (DZUP 172). (A) Dorsal view of the body, (B) ventral view of the body, (C) lateral view of the head, (D) ventral view of right hand, (E) ventral view of right foot. Horizontal scale bar = 5 mm; vertical scale bars = 2 mm.

**Figure 2:**
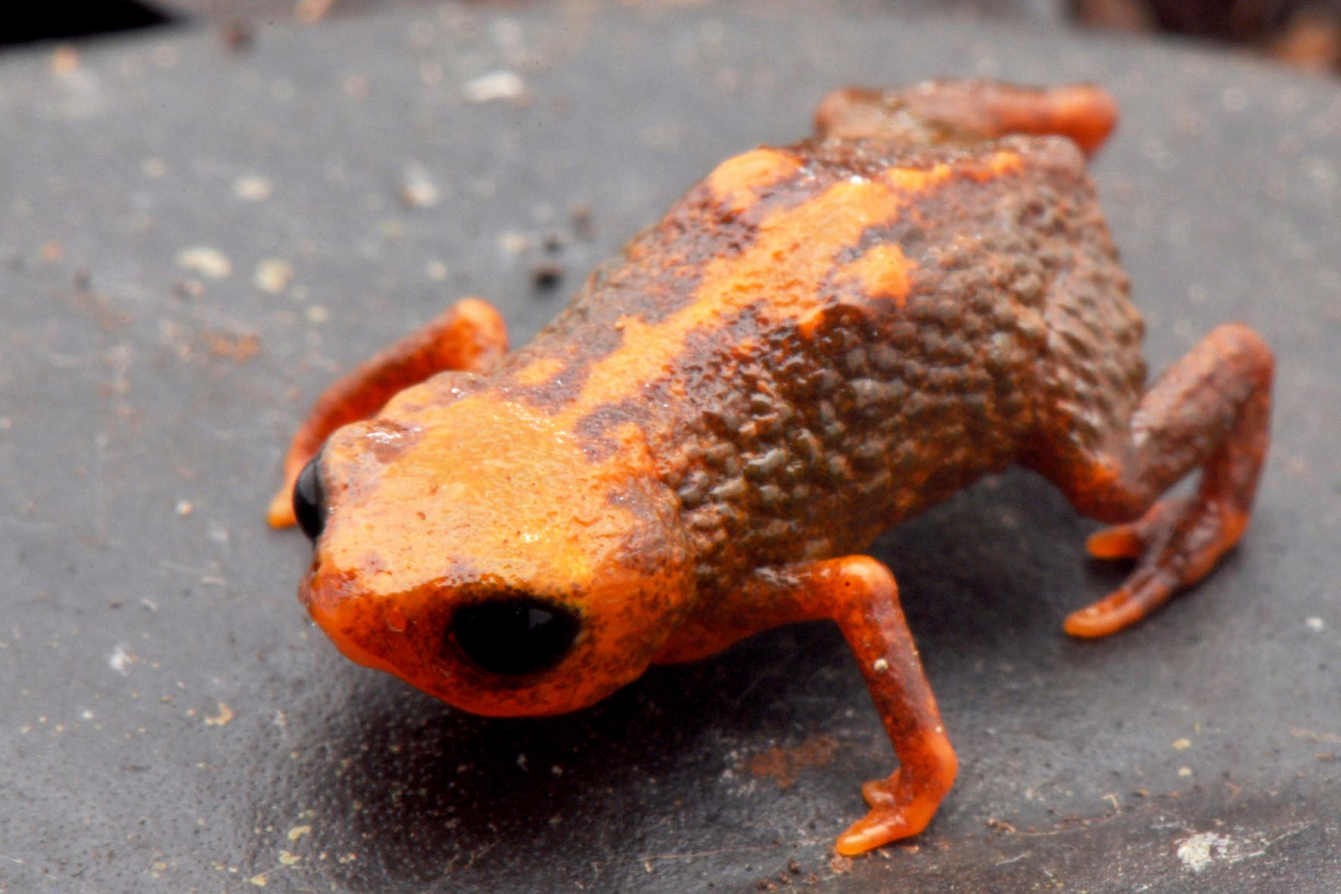
*Brachycephalus quiririensis* in life.

**Figure 3:**
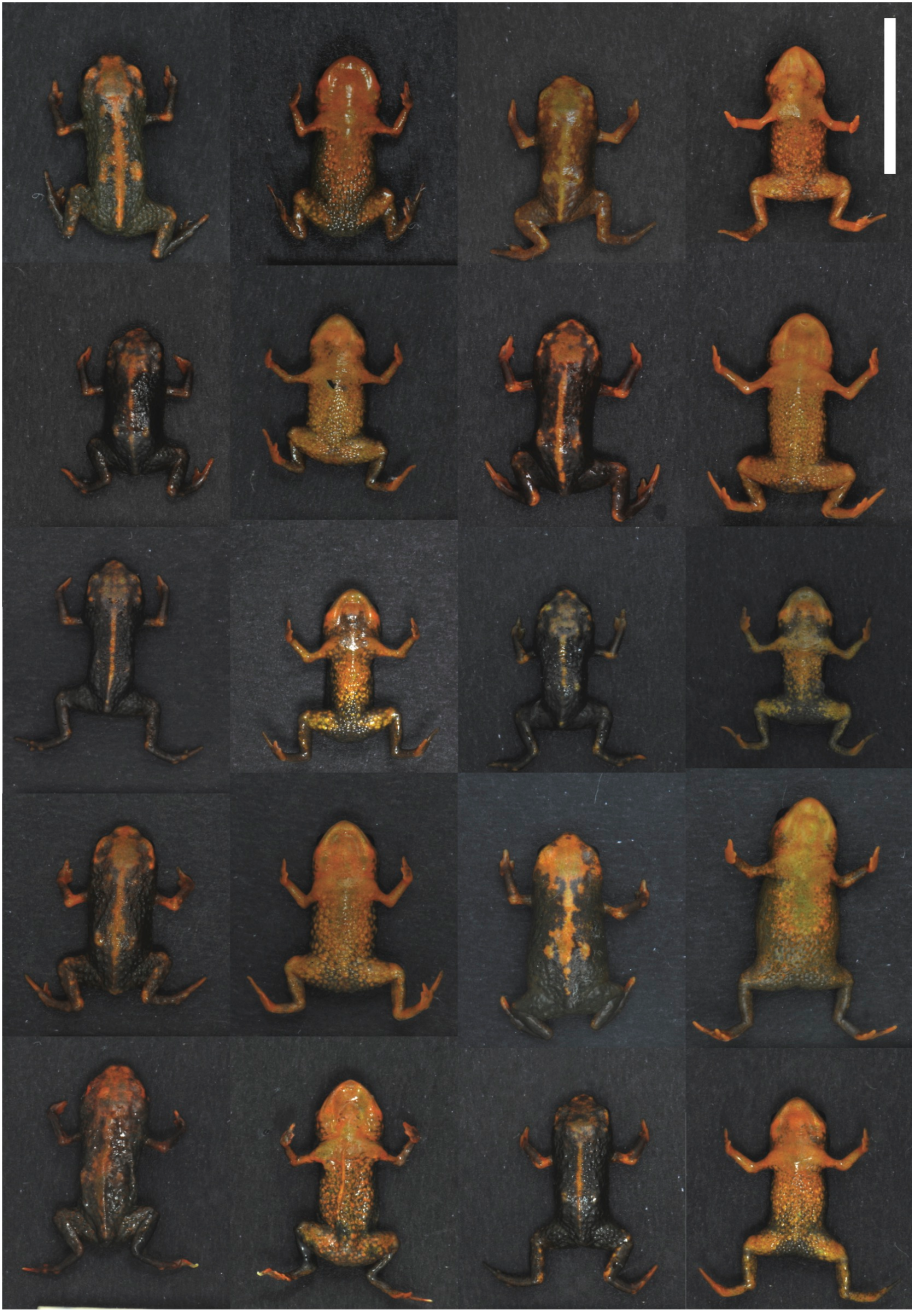
Variation in dorsal coloration in live (anesthetized) specimens of *Brachycephalus quiririensis*.

## Results

**Table:** 

*Brachycephalus****quiririensis*** sp. nov.
Urn:lsid:zoobank.org:act: A58C8AC5-6C38-447E-B2B2-3BED619FEFC8 (Figs. 1, 2 and 3)
*Brachycephalus* sp. 5 (Pie et al., 2013)

**Holotype.** DZUP 172 (Fig. 1), adult female, collected at the Serra do Quiriri, (26^°^01′17″S, 48°59′47″W at 1,263 m a.s.l.), municipality of Campo Alegre, state of Santa Catarina, southern Brazil, on 03 February 2011 by MRP, Marcos Ricardo Bornschein & Dulce Carvalho.

**Paratopotypes.** DZUP 171, 173–6, collected with the holotype. DZUP 524-30 (26°01′42″S, 48°57′54″W at 1,318 m a.s.l.), collected by MRP, LFR & Marcos Ricardo Bornschein on 20 January 2015.

**Diagnosis.***Brachycephalus quiririensis* is distinguished from all of the species in the genus by the following combination of characters: body robust, bufoniform, adult size SVL 9.9–13.1 mm, rough dorsum (Fig. 1); general background color greenish-brown, with an orange stripe that extends from the dorsum of the head and along the vertebral column; skin on dorsum of head and central body dorsum with no dermal co-ossification (Fig. 2). A representative of the *pernix* group, *B. quiririensis* is unique among other members of its species group by its brownish dorsal coloration shared *B. brunneus*, but different from that species due to the orange stripe along the vertebral column and by the greenish hue associated with the brown coloration. The new species is also distinguished from *B. ferruginus*, *B. izecksohni*, *B. pombali*, and *B. tridactylus* by having rough dorsum, with granular aspect, instead of a smooth dorsum. The new species lacks of dermal co-ossification characteristic of species of the *ephippium* group, and the bufoniform shape and larger body size of the new species distinguish it from those in the *didactylus* group, which are smaller (SVL = 8–10 mm) and have leptodactyliform body shape.

**Description of Holotype.** Body robust, bufoniform; head slightly wider than long; head length 31% of snout-vent length; snout short, with length almost equal to eye diameter, snout mucronate in dorsal view; nostrils protuberant, directed anterolaterally; canthus rostralis indistinct; lips nearly sigmoid; loreal region slightly concave; eye slightly protuberant in dorsal and lateral views; ED 34% of head length; tympanum indistinct; vocal sac not expanded externally; vocal slits present; tongue longer than wide, with posterior half not adherent to floor of mouth; choanae relatively small and round; vomerine odonthophores absent; arm and forearm relatively slender; arm approximately as long as forearm; tip of finger I and II slightly rounded, tip of finger III pointed; finger I and IV very small, vestigial; relative lengths of fingers IV <I < II < III; subarticular tubercles absent; inner and outer metacarpal tubercles absent; legs short, moderately robust; thigh length 34% of snout-vent length, tibia length 79 % of thigh length; toes II–III short, relatively distinct; toe I externally absent, toe V vestigial; relative length of toes V < II < III < IV; subarticular tubercles and inner metatarsal tubercles absent; outer metatarsal tubercle distinct, large, ovoid; rough dorsum, without co-ossifications; glandular warts large, circular to oval, present and spaced in dorsum and legs; head and arms smooth; sides of the body granular; large glandular warts circular and spaced on the sides of the body; belly granular; circular glandular warts spaced and distributed from belly to legs and arms, chin smooth.

**Coloration of Holotype.** In life, dorsum, head, sides of the body, dorsal region of arms, thighs, and legs brown; dorsal region of snout and head orange, continuing into an orange stripe along the vertebral column and forming two spots near the tips of the sacral vertebra; chin, ventral portion of arms, thighs, legs, and ventral regions of hand and feet orange; belly with orange spots against a the brownish coloration that extends from the sides of the body; iris black (Fig. 2). In preservative, the orange coloration becomes pale cream, while maintaining the brownish coloration.

**Measurements of Holotype.** SVL = 12.3 mm, HL = 3.9 mm, HW = 4.7 mm, ED = 1.3 mm, ND = 0.3 mm, IOD = 2.4 mm, IND = 1.3 mm, END = 0.7 mm, THL = 4.1 mm, TBL = 3.3 mm.

**Variation in Type Series.** Measurements and proportions of 10 adults in millimeters are (mean ± SD, with range in parentheses): SVL 11.12 ± 1.03 (9.9–13.1); HL 3.73 ± 0.31 (3.3–4.4); HW 4.23 ± 0.42 (3.7–5.0); ED 1.18 ± 0.10 (1.1–1.4); ND 0.21 ± 0.04 (0.2–0.3); IOD 2.25 ± 0.17 (2.0–2.6); IND 1.26 ± 0.11 (1.1–1.4); END 0.64 ± 0.07 (0.6–0.8); THL 3.83 ± 0.37 (3.1–4.7); TBL 3.23 ± 0.30 (2.9–3.8); THL/SVL 35 ± 2% (32–37%); TBL/THL 85 ± 6% (70–93%); HL/SVL 34 ± 1% (31–36%); ED/HL 32 ± 2% (28–34%); HL/HW 88 ± 5% (79–93%). There is variation among individuals in the breadth of the orange stripe along their backs, as well as different degrees of darkness in the brown regions (Fig. 3). Some specimens show a slight greenish hue in the brownish regions of the body.

**Etymology.** The epithet “quiririensis” is derived from the Tupi-Guarani language word “quiriri” (= silence, peace) and refers to type locality.

**Figure 4:**
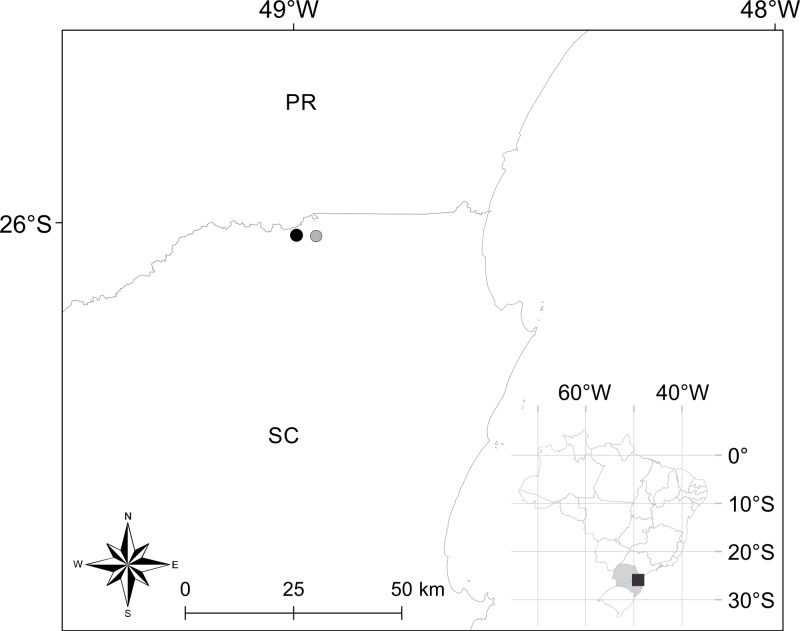
Geographical location of occurrence records of *Brachycephalus quiririensis.* Closed circle indicates the type locality. PR and SC correspond to the states of Paraná and Santa Catarina, respectively.

**Distribution.***Brachycephalus quiririensis* is known from the type locality, as well as an additional nearby site (Fig. 4) in the municipality of Garuva, state of Santa Catarina (26°01′42″S, 48°57″11″W) (Pie et al., 2013).

**Remarks.** Individuals of *B. quiririensis* were found on leaf litter of a cloud forest (“Floresta Ombrófila Densa Altomontana” *sensu* Veloso, Rangel-Filho & Lima, 1991) from 1,263 m at 1,318 m a.s.l. During two visits to the type locality, we found individuals of *B. quiririensis* hidden in the leaf litter, some of which vocalizing. The vegetation in the area has experienced severe disturbances in recent years, particularly fire and extensive cattle ranching. Moreover, two highly endemic bufonid species of the genus *Melanophryniscus* are found in the region (Pie et al., 2013; Bornschein et al., 2015, unpublished data), one of which occurring syntopically with *B. quiririensis*. Strong measures should be taken by the environmental protection agency of the State of Santa Catarina to ensure the long-term preservation of these species. In addition, *B. quiririensis* should be integrated into the “Plano de Ação Nacional para a Conservação dos Anfíbios e Répteis Ameaçados da Região Sul do Brasil,” an initiative organized by the Instituto Chico Mendes/Ministry of the Environment that seeks to organize conservation initiatives for the conservation of the herpetofauna of southern Brazil.

## Supplemental Information

10.7717/peerj.1179/supp-1 Appendix S1 Examined specimens

## Additional Information and Declarations

### Field Study Permissions

The following information was supplied relating to field study approvals (i.e., approving body and any reference numbers): Collection permits for this study were issued by ICMBIO (SISBIO 1911426).

### New Species Registration

The following information was supplied regarding the registration of a newly described species: Species info: *Brachycephalus quiririensis* urn:lsid:zoobank.org:act:A58C8AC5-6C38-447E-B2B2-3BED619FEFC8

## References

[ref-1] Alves ACR, Ribeiro LF, Haddad CFB, Reis SF (2006). Two new species of *Brachycephalus* (Anura: Brachycephalidae) from the Atlantic Forest in Paraná State, southern Brazil. Herpetologica.

[ref-2] Carnaval AC, Hickerson MJ, Haddad CF, Rodrigues MT, Moritz C (2009). Stability predicts genetic diversity in the Brazilian Atlantic forest hotspot. Science.

[ref-3] Carnaval AC, Waltari E, Rodrigues MT, Rosauer D, VanDerWal J, Damasceno R, Prates I, Strangas M, Spanos Z, Rivera D, Pie MR, Firkowski CR, Bornschein MR, Ribeiro LF, Moritz C (2014). Prediction of phylogeographic endemism in an environmentally complex biome. Proceedings of the Royal Society B: Biological Sciences.

[ref-4] Clemente-Carvalho RBG, Klaczko J, Perez SI, Alves ACR, Haddad CFB, dos Reis SF (2011). Molecular phylogenetic and phenotypic diversity in miniaturized toadlets, genus *Brachycephalus* (Amphibia: Anura: Brachycephalidae). Molecular Phylogenetics and Evolution.

[ref-5] Da Silva JMC, Cardoso de Sousa M, Castelletti CH (2004). Areas of endemism for passerine birds in the Atlantic forest, South America. Global Ecology and Biogeography.

[ref-6] De Queiroz K (2005). Ernst Mayr and the modern concept of species. Proceedings of the National Academy of Sciences of the United States of America.

[ref-7] Howell JT (1947). Mono Mesa—Sierra Sky Island. Sierra Club Bulletin.

[ref-8] McCormack JE, Huang H, Knowles LL, Gillespie RG, Clague D (2009). Sky islands. Encyclopedia of Islands.

[ref-9] Myers N, Mittermeier RA, Mittermeier CG, Da Fonseca GA, Kent J (2000). Biodiversity hotspots for conservation priorities. Nature.

[ref-10] Napoli MF, Caramaschi U, Cruz CAG, Dias IR (2011). A new species of flea-toad, genus *Brachycephalus* Fitzinger (Amphibia: Anura: Brachycephalidae), from the Atlantic rainforest of southern Bahia, Brazil. Zootaxa.

[ref-11] Padial JM, Grant T, Frost DR (2014). Molecular systematics of terraranas (Anura: Brachycephaloidea) with an assessment of the effects of alignment and optimality criteria. Zootaxa.

[ref-12] Pie MR, Meyer ALS, Firkowski CR, Ribeiro LF, Bornschein MR (2013). Understanding the mechanisms underlying the distribution of microendemic montane frogs (*Brachycephalus* spp., Terrarana: Brachycephalidae) in the Brazilian Atlantic Rainforest. Ecological Modelling.

[ref-13] Pombal JP, Wistuba EM, Bornschein MR (1998). A new species of brachycephalid (Anura) from the Atlantic Rain Forest of Brazil. Journal of Herpetology.

[ref-14] Ribeiro LF, Alves ACR, Haddad CFB, Reis SF (2005). Two new species of *Brachycephalus* Guenther, 1858 from the state of Parana, southern Brazil (Amphibia, Anura, Brachycephalidae). Boletim do Museu Nacional, Nova Série, Zoologia.

[ref-15] Ribeiro LF, Bornschein MR, Belmonte-Lopes R, Firkowski CR, Morato SAA (2015). Seven new microendemic species of *Brachycephalus* (Anura: Brachycephalidae) from southern Brazil. PeerJ.

[ref-16] Sigrist MS, Carvalho CJBD (2008). Detection of areas of endemism on two spatial scales using Parsimony Analysis of Endemicity (PAE): the Neotropical region and the Atlantic Forest. Biota Neotropica.

[ref-17] Veloso HP, Rangel-Filho ALR, Lima JCA (1991). Classificação da vegetação brasileira, adaptada a um sistema universal.

