# Supplementary material for: A new species of Brachycephalus (Anura: Brachycephalidae) from the Quiriri mountain range of southern Brazil
Source: PeerJ. 2015 Aug 13;3:e1179. doi: 10.7717/peerj.1179 (PMC4558078; doi:10.7717/peerj.1179)
Supplement: Appendix S1 [file peerj-03-1179-s001.docx]

Appendix I. Examined specimens.

*Brachycephalus auroguttatus*. SANTA CATARINA: Pedra da Tartaruga, municipality of Garuva DZUP 373-5, 376-85, 387-89.

*Brachycephalus boticario*. SANTA CATARINA: Morro do Cachorro, on the border between the municipalities of Blumenau, Gaspar, and Luiz Alves. DZUP 414-5, 438-40, 444-5.

*Brachycephalus brunneus*. PARANÁ: Caratuva, Serra dos Órgãos, municipality of Campina Grande do Sul MHNCI 1919-20, MNRJ 40289-91 (paratypes).

*Brachycephalus didactylus*. RIO DE JANEIRO: municipality of Engenheiro Paulo de Frontin ZUEC 1133, 10825, 1132, MZUSP 94621; Sacra Família do Tinguá, municipality of Engenheiro Paulo de Frontin MZUSP 13613-20, 64810-1.

*Brachycephalus ephippium*. RIO DE JANEIRO: Parque Nacional Serra dos Órgãos, MZUSP 104140-7.

*Brachycephalus ferruginus*. PARANÁ: Olimpo (25°27'03"S, 48°54'59"W), Serra do Marumbi, municipality of Morretes MHNCI 125, 128.

*Brachycephalus fuscolineatus*. SANTA CATARINA: Morro do Baú, municipality of Ilhota DZUP 158-60, 401-5.

*Brachycephalus hermogenesi*. SÃO PAULO: Ubatuba ZUEC 9715-21, 9723-5. Reserva Florestal de Morro Grande, municipality of Cotia MZUSP 132257-63.

*Brachycephalus izecksohni*. PARANÁ: Torre da Prata, Serra da Prata, boundary of the municipalities of Morretes, Paranaguá, and Guaratuba CFBH 7381-2, 7384 (paratypes).

*Brachycephalus leopardus*. PARANÁ: Serra do Araçatuba, municipality of Tijucas do Sul DZUP 478-492.

*Brachycephalus mariaeterezae*. SANTA CATARINA: Reserva Particular do Patrimônio Natural Caetezal, top of the Serra Queimada, municipality of Joinville MHNCI 9811-2, DZUP 372, 393-9 (paratypes).

*Brachycephalus nodoterga*. SÃO PAULO: Santana de Parnaíba, MZUSP 147711-6.

*Brachycephalus pernix*. PARANÁ: Anhangava, Serra da Baitaca, municipality of Quatro Barras CFBH 2597-8 (paratypes), MHNCI 1818-9 (paratypes) 1820, 3000-4 (paratypes), MNRJ 17349 (holotype), ZUEC 9433-7 (paratypes), DZUP 539-55.

*Brachycephalus olivaceus.* SANTA CATARINA: Reserva Particular do Patrimônio Natural Caetezal, top of the Serra Queimada, municipality of Joinville MHNCI 9813-8, DZUP 371.

*Brachycephalus pombali.* PARANÁ: Morro dos Padres, Pico da Igreja (25°39'S, 48°51'W), municipality of Guaratuba CFBH 8042 (holotype), 8043-53 (paratypes).

*Brachycephalus tridactylus*. PARANÁ: Serra do Morato, Guaraqueçaba, DZUP493-7.

*Brachycephalus verrucosus*. SANTA CATARINA: Morro da Tromba, municipality of Joinville MHNCI 9819-20, DZUP 464-78.
